# A Rare Case of Pilomatrixoma (Calcifying Epithelioma of Malherbe) of Parotid Space Masquerading as Salivary Gland Tumor

**Published:** 2016

**Authors:** Manas Bajpai, Manika Arora, Betina Chandolia

**Affiliations:** 1 *Dept of Oral and maxillofacial Pathology, NIMS Dental College, Jaipur*

**Keywords:** Pilomatrixoma, calcifying epithelioma of malherbe, parotid space, salivary gland tumor

**Dear Editor-in-Chief**

Pilomatrixoma is a benign tumor of hair follicle first described as calcifying epithelium (1). It accounts for 1% of all the benign skin tumors (2). It is a slow-growing, firm, dermal or subcutaneous neoplasm, usually measuring fewer than 3 cm in diameter. Pilomatricomas are considered benign; rarely recur after surgical excision (3). However, cases of pilomatrixoma with local recurrence and local invasiveness have been reported in the literature. 

Microscopically, the tumor is characterized by proliferation of basalloid cell masses arranged haphazardly throughout the tumor with varying degree of cytological atypia with eosinophilliccornified material and abundant shadow cells (4).We hereby present a case of pilomatrixoma arising in a parotid space of a 28 yrold lady.

A 28 yr old woman presented to the Department of Oral Medicine and Radiology, NIMS Dental College Jaipur (India) in December 2015 with a chief complaint of a swelling and pain on her left subauricular region, the lesion had disappeared and relapsed several times over a one year of duration (Fig. 1). On palpation lesion,it was firm and movable with tenderness. Cervical lymph nodes were non – palpable. Ultrasonography revealed a patchy radiopaque lesion of the parotid space (Fig. 2). Clinical differential diagnoses of pleomorphic adenoma, pilomatrixoma and neurogenic tumors with dystrophic calcification were considered. Informed consent of the patient was obtained. A surgical procedure was performed under general anesthesia. The mass was exposed and removed along with surrounding tissue by extracapsular enucleation. The excised specimen was sent to the Department of Oral and Maxillofacial Pathology NIMS Dental College (Jaipur). The gross specimen showed a firm gritty cut (Fig. 3).

Histological examination revealed a peripheral layer of basophilic cells resembling epithelium basaloid cells. The connective tissue stroma was loose and fibrilar (Fig. 4). A central area exhibited collection of numerous nucleus free cells with diaphanous cytoplasm resembling shadow cells (Fig. 5) with dilated blood vessels and areas of eosinophiliccornified material resembling calcifications (Fig. 6). Hence, the final diagnosis of pilomatrixoma was rendered. The patient’s follow up period of 2 yrwas uneventful.

Pilomatrixomas are rare benign low growing tumors of skin, chiefly affects pediatric population. Clinically, they present as well circumscribed, solitary, painless, subcutaneous tumors. The lesions grow slowly, without itching or other symptoms (5). The skin over the neoplasm may present normal appearance or changes in color from pale to red or bluish depending on the secondary inflammation (1-3). The lesions are fixed to the overlying skin, but mobile in relation to deep planes. Epithelial thinning or even ulceration may occur. Pilomatrixomas show predilection for the face, but may be present in any region of the body, except in the palms or soles. Pilomatrixomas are rarely found on parotid space only few cases have been reported (4). Pilomatricoma is diagnosed by clinical examination (1, 3, 4). “The consistency of the lesion varies considerably, depending on the degree of calcification. Ultrasound examination may be helpful in diagnosis” (6).

Histological features of pilomatrixomas are typical and shows haphazardly arranged basalloid cells throughout the lesion with areas of shadow cells and abundant calcified areas (4).

**Fig 1:**
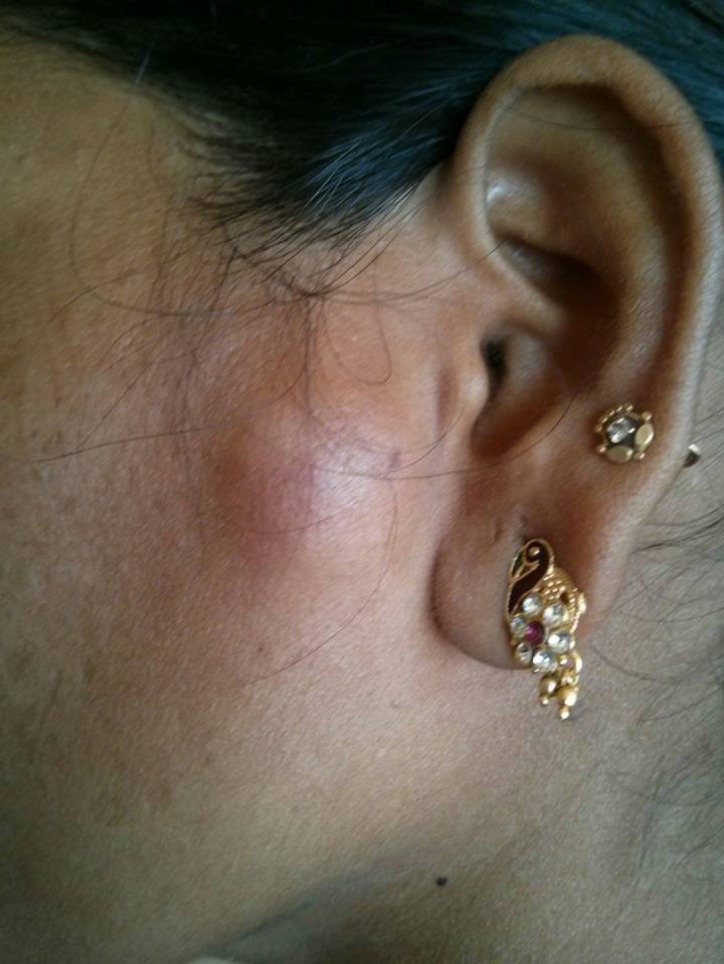
Swelling of the subauricular area

**Fig 2:**
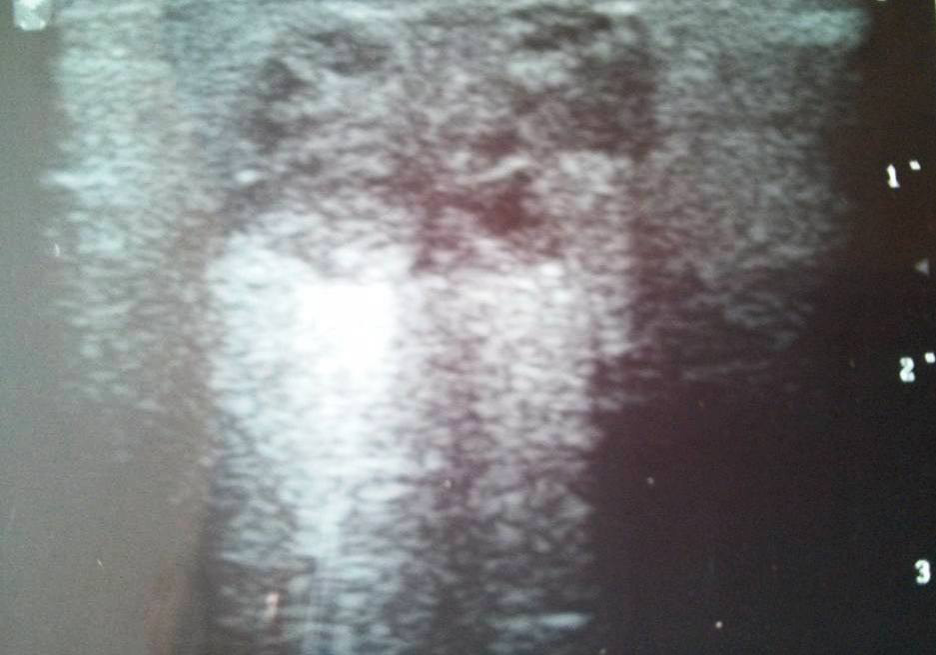
Radiograph reveals calcifications of the parotid space

**Fig 3:**
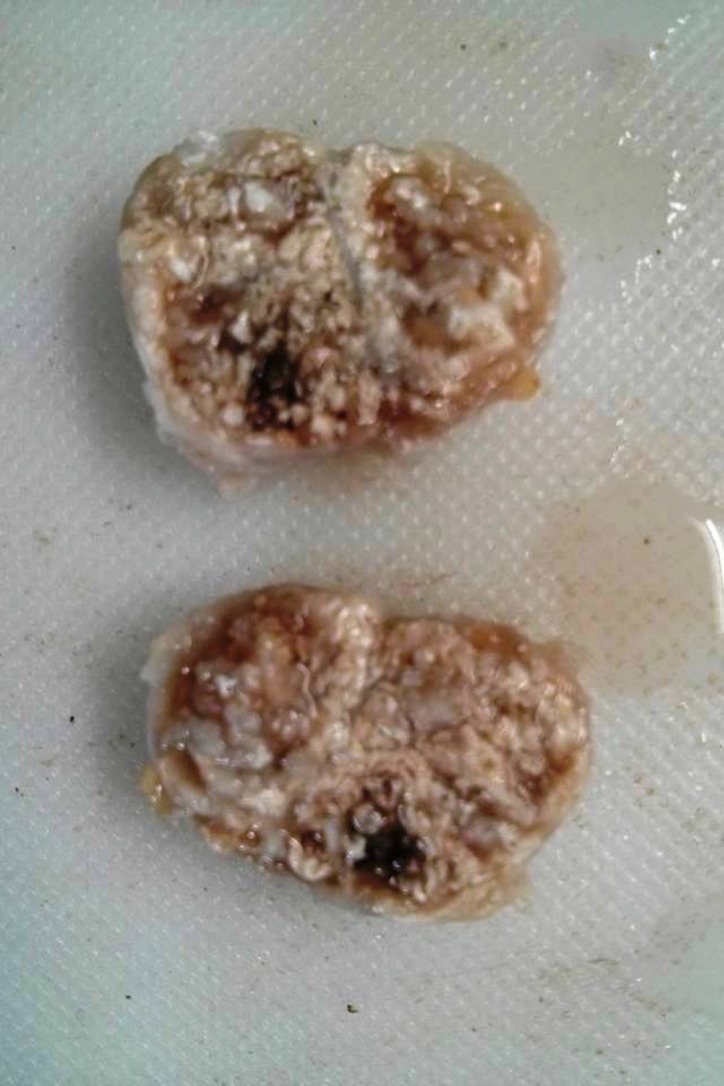
Cut section of the specimen

**Fig 4:**
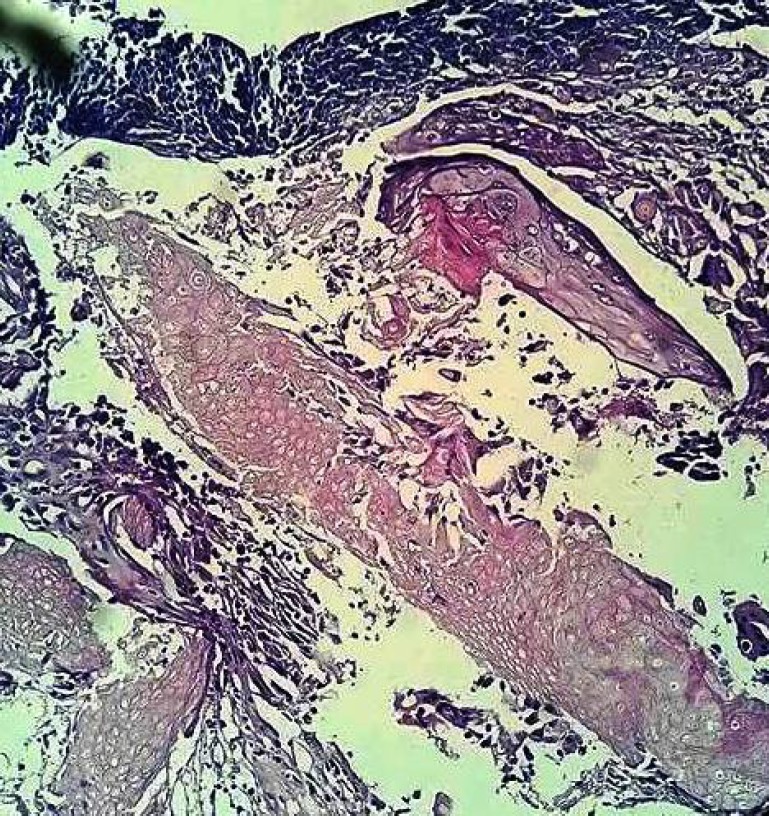
Areas of basaloid squamous cells (Hematoxilin and Eosin stain X40

**Fig 5:**
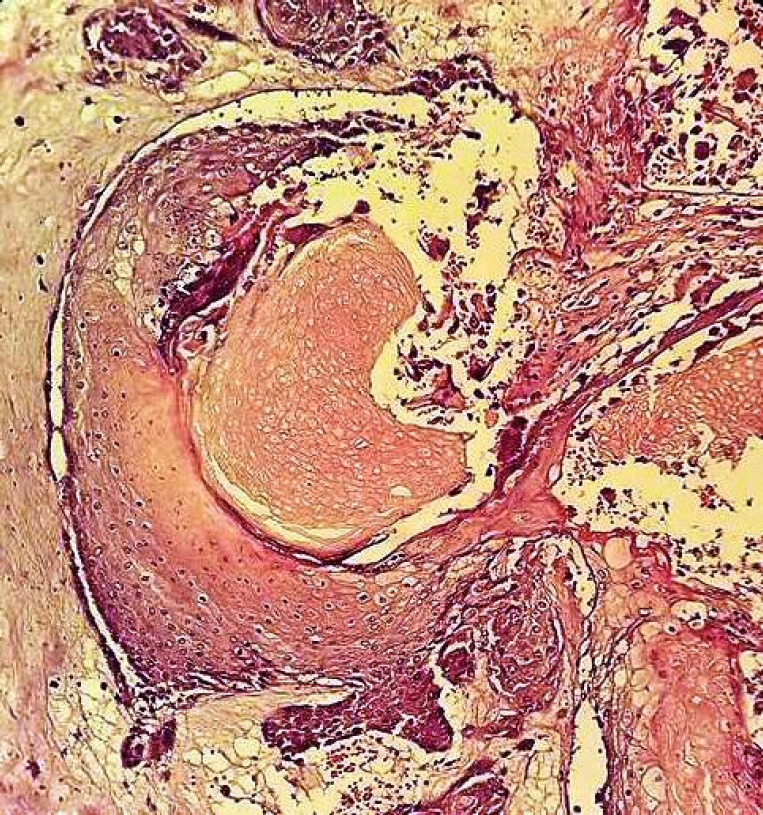
Areas of haphazardly arranged epithelium basaloid cells and shadow cells (Hematoxilin and Eosin stain X40

**Fig 6:**
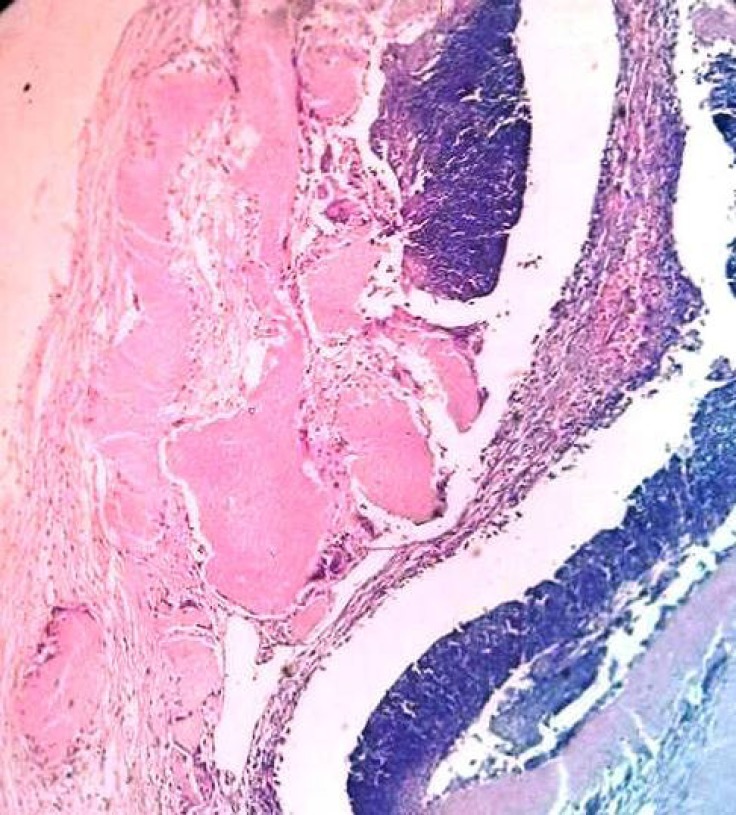
Areas of eosinophiliccornified materials (Hematoxilin and Eosin stain X20

## Conflict of Interest:

The authors declare that there is no Conflict of Interests.
